# Prevalence of Independence at Home–Qualifying Beneficiaries in Traditional Medicare, 2014-2021

**DOI:** 10.1001/jamanetworkopen.2024.21102

**Published:** 2024-07-11

**Authors:** Tom Lally, Emily Johnson, Konstantinos E. Deligiannidis, George Taler, Peter Boling, Aaron Yao, Joanna Kubisiak, Angelina Lee, Bruce Kinosian

**Affiliations:** 1 Bloom Health, Denver, Colorado; 2 Department of Family Medicine, Donald and Barbara Zucker School of Medicine at Hofstra/Northwell, Hempstead, New York; 3 Geriatrics and Senior Services, Medstar Health, Baltimore, Maryland; 4 Division of Geriatrics, Virginia Commonwealth University, Richmond; 5 Section of Geriatrics, University of Virginia School of Medicine, Charlottesville; 6 Home Centered Care Institute, Schaumberg, Illinois; 7 Westat, Rockville, Maryland; 8 Division of Geriatrics, Perelman School of Medicine, University of Pennsylvania, Philadelphia; 9 Leonard Davis Institute of Health Economics, University of Pennsylvania, Philadelphia

## Abstract

**Importance:**

The Centers for Medicare & Medicaid Services Innovation Center Independence at Home (IAH) demonstration, a test of home-based primary care operating in a value-based shared-savings payment model, ended December 2023 after a decade of consistently showing savings to Medicare. It is important to assess whether high-need, IAH-qualified beneficiaries continue to pose a growing challenge to traditional Medicare (TM) or if Medicare Advantage (MA), with programmatic features favorable to caring for this subset of the general Medicare population, can disproportionately provide such care.

**Objective:**

To examine the size and share of IAH-qualified beneficiaries in TM and MA.

**Design, Setting, and Participants:**

This cohort study used all Medicare claims data and MA encounter data for 2014 and 2021. IAH qualifying criteria were applied to the TM populations enrolled in Parts A and B in 2014 and 2021, and to MA enrollees in 2021. Growth in the number of IAH-qualified TM beneficiaries from 2014 to 2021 was calculated, and the proportions and numbers of IAH-qualified enrollees in the total 2021 TM and MA populations were compared. Data were analyzed between April and June 2023.

**Main Outcomes and Measures:**

The number and share of beneficiaries meeting IAH criteria in TM and MA; the share of TM spending among IAH-qualified beneficiaries.

**Results:**

Among 64 million Medicare beneficiaries in 2021, there were 30.55 million beneficiaries in TM with Parts A and B coverage, down from 33.82 million in 2014. The number of IAH-qualified beneficiaries in TM grew 51%, from 2.16 million to 3.27 million, while their proportionate share in TM grew 67% from 6.4% to 10.7% of TM between 2014 and 2021. IAH-qualified beneficiaries represented $155 billion in 2021 Medicare Parts A and B spending, 44% of all TM spending, up from 29% of total spending in 2014. In 2021, 2.15 million IAH-qualified beneficiaries represented 8.0% of Medicare Advantage enrollees. Combining TM and MA, 5.42 million IAH-qualified beneficiaries represented 9.3% of all Medicare beneficiaries, with 3.27 million (60.3%) being insured by TM.

**Conclusions and Relevance:**

In this cohort study of IAH-qualified Medicare beneficiaries, the share of IAH-qualified beneficiaries in TM grew between 2014 and 2021, with 60% of Medicare high-need beneficiaries accounting for 44% of TM spending. The Centers for Medicare & Medicaid Services should continue to operate value-based programs like IAH that are specifically designed for these high-needs individuals.

## Key Points

**Question:**

Has there been growth in the high-needs, Independence at Home (IAH)-qualified population in traditional Medicare (TM) between 2014 and 2021, and how does the size of the IAH-qualified population in TM compare with Medicare Advantage (MA)?

**Findings:**

In this cohort study, the IAH-qualified beneficiary population in TM grew from 2.16 million in 2014 (6.4% of TM) to 3.27 million in 2021 (10.7% of TM). In TM, the IAH-qualified population accounted for 44% of Parts A and B TM spending in 2021 vs 29% in 2014; the proportion of the IAH-qualified beneficiaries was 33% larger in TM than in MA.

**Meaning:**

These results suggest that the IAH-qualified subset of the TM population and their share of TM spending has increased; MA is not disproportionately caring for such high-need beneficiaries, despite programmatic features to facilitate such care, an observation that reinforces the need for accessible high-needs clinical programs for IAH-qualified beneficiaries in TM.

## Teaser

This cohort study assesses the number of Medicare beneficiaries who qualify for the Independence at Home program in traditional Medicare vs Medicare Advantage from 2014 to 2021.

## Introduction

The decade-long Independence at Home (IAH) demonstration (2012-2023) demonstrated the efficacy of delivering home-based primary care (HBPC) to high-need, high-cost traditional Medicare (TM) beneficiaries.^1^ The IAH target population had serious chronic health problems, hospitalization followed by use of Medicare postacute benefits, and had difficulty accessing routine medical care because of ADL impairments. In 2021, IAH participant sites achieved publicly reported Medicare cost savings of 21%, comparable with HBPC providers in a successor demonstration for similar beneficiaries, High Needs Direct Contracting.^2^ The IAH demonstration evaluated if the HBPC model, which includes a mobile interdisciplinary team, proactive outreach, and high visit frequency, combined with financial incentives to limit unnecessary use of facility-based acute care, could improve health outcomes while lowering the total cost of care. Recipients of HBPC were less likely to be hospitalized and tended to spend more total days at home than similar patients who did not receive HBPC.^1^ Through year 8 of the demonstration, total cost of care was $229 million ($3165 per beneficiary per year) less than a carefully matched comparison cohort, and $209 million less than Centers for Medicare & Medicaid Services’ (CMS) actuarially predicted costs. The net savings to CMS, after accounting for the $81 million invested in the IAH participant sites through shared savings payments, were over $148 million (eTable 1 in Supplement 1).

The IAH criteria are notable for their clinical transparency and ability to identify a high-cost segment of the fee-for-service (FFS) Medicare population. IAH-qualified Medicare beneficiaries must have 2 or more dependencies in basic activities of daily living (ADLs), 2 or more significant chronic medical conditions, and both a nonelective hospitalization and postacute care utilization in the prior 12 months. At demonstration inception in 2012, IAH-qualified individuals comprised 6% of traditional Medicare beneficiaries yet accounted for 29% of Medicare spending, 24% of hospitalizations, 23% of deaths, and 38% of long-term nursing home admissions.^3^

The other major payor for Medicare-eligible US citizens is Medicare Advantage (MA), which has grown dramatically since 2012, from 27% of the total Medicare population to 50% in 2023.^4^ MA has greater flexibility than TM to innovate and provide care management and enhanced services to high-need beneficiaries, characteristics that value-based programs like IAH seek to harness.^5^ Beyond the required usual care services, the large number of MA plans vary in their programmatic offerings and geographic coverage. As IAH has just ended, we used Medicare data to determine the size and share of IAH-qualified beneficiaries in TM and MA, looking to assess how best to meet the needs of this important group of beneficiaries.

## Methods

This cohort study was exempt from institutional review board review with the CMS Virtual Research Data Center because it was performed for operational purposes, and did not require informed consent because all privacy restrictions were followed, including cell size limits. We followed the Strengthening the Reporting of Observational Studies in Epidemiology (STROBE) reporting guideline.

### Statistical Analysis

Using 100% Medicare claims, TM beneficiaries who may be classified as aged or disabled in 2021 with Medicare Parts A and B were assessed for meeting IAH criteria (hospitalization and postacute care in the prior 12 months, 2 or more chronic conditions that can impact function, 2 or more ADL impairments), both at the start of 2021 and during the subsequent 12 months. ADL impairments were clinician-reported in IAH; to create a proxy for this criterion, we used a claims-based frailty index, the JEN Frailty Index (JFI), which uses diagnoses and utilization infovrmation from claims as indicators of frailty. We set the JFI score threshold at 6 or higher.^6^ In 2021, an IAH-qualified population in TM identified using a JFI score of 6 or higher had equivalent mortality, cost, hospitalization, and long-term institutionalization rates to the population identified by the high-needs criteria currently used in CMS’s High Needs Accountable Care Organization (ACO) REACH (Realizing Equity, Access, and Community Health) model.^6^

We then applied the same IAH criteria to the 2014 5% sample to identify the number and proportion of IAH-qualified beneficiaries in 2014 (we did not have access to the 100% sample for 2014). To calculate absolute national IAH-qualified numbers and costs in 2014, we extrapolated from the 5% sample to 100%. We validated our extrapolation methodology by applying it to 2021, for which we had both the 5% and 100% samples.

We also used the same IAH criteria to identify IAH-qualified MA beneficiaries using 2020-2021 MA encounter data. To operationalize the ADL criterion, we used the Kim Claims-Based Frailty Index (CFI),^7^ using a threshold of 0.20 that is equivalent to a JFI score of 6 and higher as well as the CMS High Needs criteria in TM (eAppendix 1 in Supplement 1), which was equivalent to the JFI 6-point threshold in 2014.^8^ We used the CFI for the MA population instead of the JFI because of license limitations with use of the JFI.

We measured growth in IAH-qualified beneficiaries comparing the 2014 and 2021 TM Medicare claims, using a JFI threshold of 6. We then compared the prevalence of IAH-qualified in 2021 using the 2020-2021 TM Medicare claims and MA encounter data, at the national and state level.

Data analyses were descriptive, with no statistical threshold for significance required. Analyses were performed with Excel version 16.83 (Microsoft) with exported data with SAS (SAS Institute Inc) for data assembly within the VDRC.

## Results

In 2021, using the 100% Medicare claims, there were 3.27 million IAH-qualified TM beneficiaries out of 30.55 million total TM beneficiaries (10.7%) (eTable 2 in Supplement 1). They accounted for $155 billion of the $350 billion in Medicare FFS Parts A and B spending (44%) (eTable 3 in Supplement 1). The number and share of TM beneficiaries that are IAH-qualified varied by state, from 4.6% (4176 of 90 953 beneficiaries) in Alaska to 14.4% (43 100 of 299 427 beneficiaries) in Connecticut—generally lower in less densely populated states (eTable 2 in Supplement 1).

**Figure 1.**
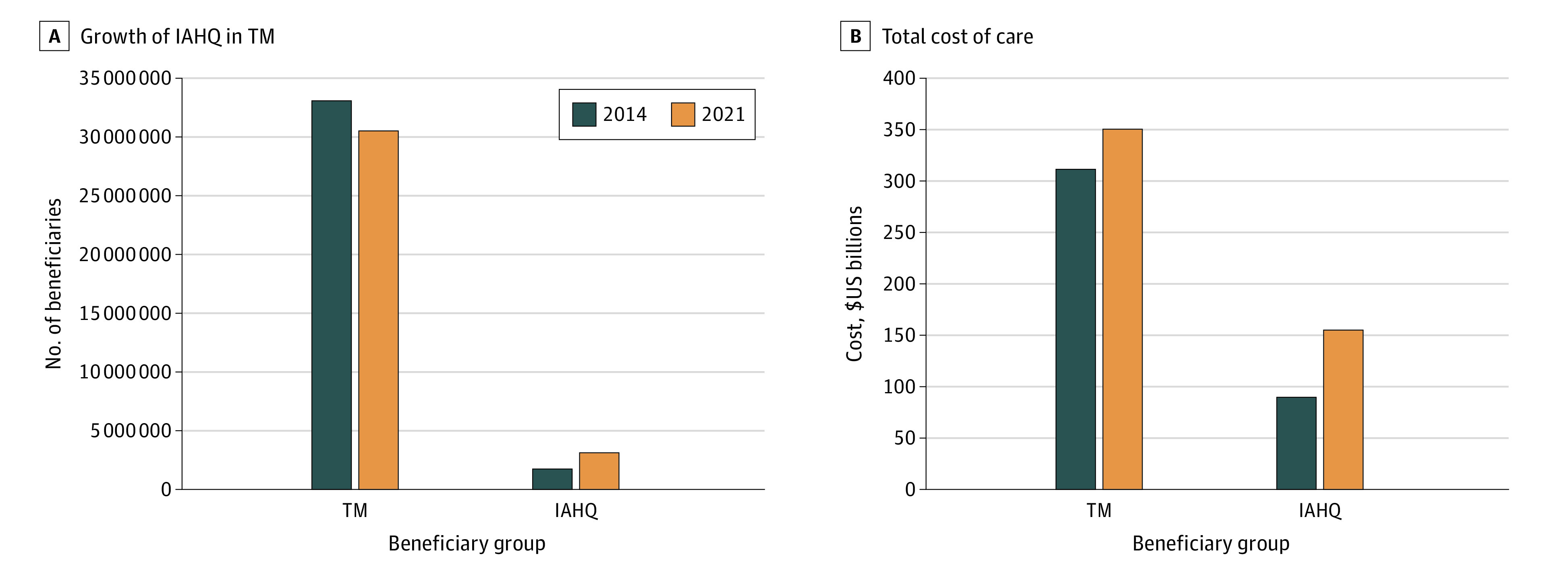
Change in Traditional Medicare (TM) and Independence at Home-Qualified Medicare (IAHQ) Beneficiaries Between 2014 and 2021

**Figure 2.**
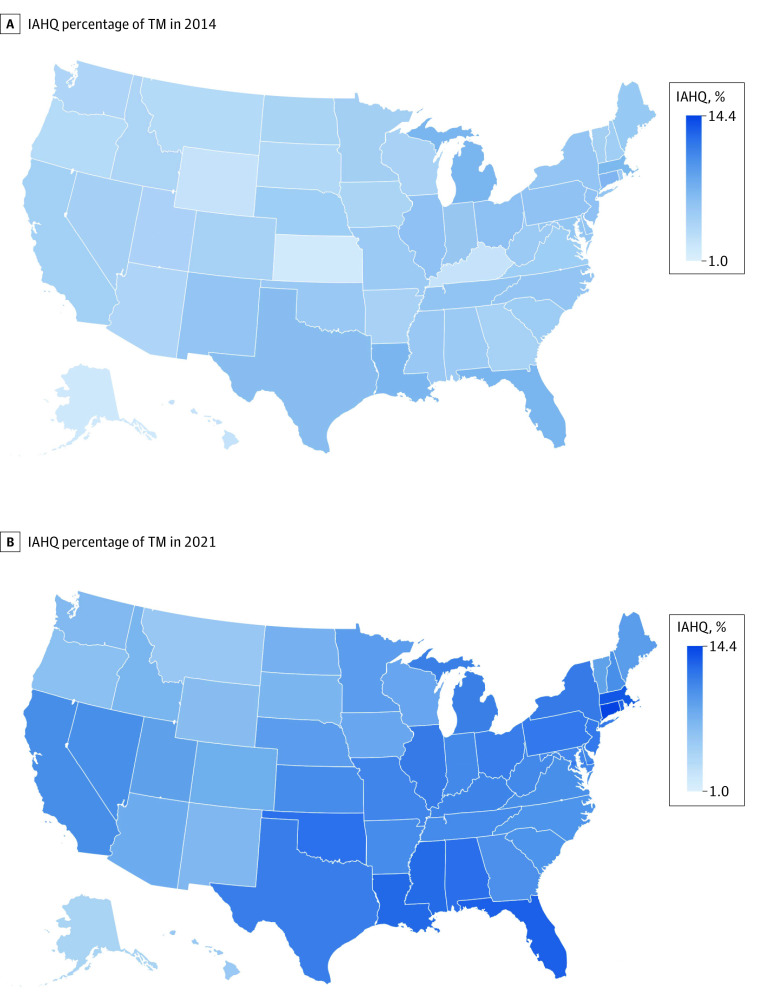
Change in Independence at Home-Qualified Medicare (IAHQ) Beneficiaries Between 2014 and 2021, By State Heat map of the share of traditional Medicare represented by IAHQ beneficiaries in 2014 and 2021, showing the explosive growth of this high needs population over 7 years.

From 2014 to 2021, the number of IAH-qualified beneficiaries grew by 1.11 million (51.4%), from 2.16 million (6.4% of all TM beneficiaries) to 3.27 million (10.7% of TM beneficiaries). Our estimate based on the 2021 5% sample was similar to the number obtained from the full 2021 sample (1.13 million vs 1.11 million), suggesting that the 5% sample can reasonably be used to extrapolate to the full population, as we did for 2014 due to data availability. Over the same time period, the TM population declined by 3.33 million (−9.8%) despite the overall Medicare population growing by 15%, reflecting more people joining MA. In TM, the growth in the share of IAH-qualified beneficiaries (67.2%) exceeded the growth in the number of IAH-qualified beneficiaries (51.1%), suggesting increasing concentration of IAH-qualified beneficiaries in TM (Figure 1A). The $40 billion growth in TM Parts A and B spending over this interval was likewise concentrated among the 3.27 million IAH qualified ($65 billion). Among the 27.22 million TM beneficiaries not qualified for IAH, spending declined (−$25 billion) (Figure 1B). The growth of the IAHQ population was broad, covering every region of the country, although with more noticeable growth in California and Nevada, the South, and the Northeast (Figure 2).

We compared the share of IAH-qualified beneficiaries in TM and MA using a JFI threshold of 6 and a CFI equivalent of 0.20. By these standards, 2.15 million (8.0%) of MA beneficiaries met IAH qualification nationally, ranging from 2.2% in North Dakota (680 of 31 212 MA beneficiaries) to 10.5% in Massachusetts (41 599 of 401 151 MA beneficiaries) (eTable 2 in Supplement 1). Combining the adjusted MA and TM populations, there were 5.42 million IAH-qualified beneficiaries in 2021, or 9.3% of the Medicare population, with 3 215 057 (60.3%) of them in TM.

## Discussion

The 7-year, 50% increase of high-need beneficiaries in TM (in this analysis, IAH-qualified beneficiaries), and the increase in their share of TM parts A and B spending, highlights the need for effective TM programs to meet the needs of this critical population. The finding that the share of IAH-qualified beneficiaries is higher in TM than MA is consistent with findings from other work: longitudinal studies from the Medicare Current Beneficiary Survey^9^ and analysis of Medicare claims^10^ have suggested a greater concentration of complex and frail beneficiaries in TM.^11^ Both beneficiary choice (beneficiaries who switch from MA to TM are more likely to have functional dependencies^12^) and provider network selection (practices with more complex and expensive panels are less likely to be included in MA networks^13,14^) may contribute to the finding.

If high-need beneficiaries are disproportionately represented in TM, it will be important for CMS to operate value-based programs like IAH within TM that are specifically designed for high-needs individuals. Over the same period that IAH-qualified beneficiaries in TM grew by 1.11 million, the number of IAH-qualified beneficiaries who received HBPC services under fee-for-service TM grew by less than 250 000.^15^ High-needs populations and the health care professionals that care for them have distinctive characteristics that are often not adequately addressed by large one-size-fits all programs that are designed for the general Medicare population, such as standard ACOs.

The clinical care model is as important as solving the problem of patient selection, risk-adjustment, and funding. The function-limited and thus relatively immobile high-needs population that enrolled in the IAH demonstration are likely to benefit most from health care managed by mobile medical teams, organized for the purpose of providing care in the home. The limitations of the usual care office-based practice model mitigated by home-based primary care are in several areas: (1) need to provide timely access; (2) time set aside from the busy day for intensive case management work; (3) presence of an interprofessional care team; (4) familiarity with the home environment; and (5) the establishment of trust in the health care team.

### Features for Medicare Programs to Serve High-Needs Beneficiaries

To sustainably support health care professionals that effectively serve high-needs beneficiaries in TM, we recommend programs have the following key features.

#### Concurrent Risk Adjustment

Prospective risk adjustment models, which predict beneficiary costs for future years based on their health status in previous years, can be relatively accurate for an average risk beneficiary population, but they significantly underestimate costs for high-risk beneficiaries. As a result, participants in accountable care programs that use prospective risk adjustment, such as the Medicare Shared Savings Program (SSP) or MA, are underbenchmarked for high-need beneficiaries, requiring subsidizing their care from lower-risk subgroups or avoiding high-need beneficiaries altogether. The growing share of high-need beneficiaries in TM, driven by the relative shift of non–high-need beneficiaries to MA, also challenges ACOs who face an increasing demand to cross-subsidize care for high-need beneficiaries. Concurrent benchmarking, which has been implemented in the High Needs track of the ACO REACH model run by the Center for Medicare and Medicaid Innovation, is better suited to predicting costs for high need beneficiaries, similar to ad hoc adjustments CMS made to the prospective hierarchical condition category model for Program of All-Inclusive Care for the Elderly (PACE)^16^ and IAH^17^ (eAppendix 2 in Supplement 1).

#### Smaller Beneficiary Panel Minimums

As exemplified by PACE^16^ and the HBPC programs that participated in the IAH demonstration,^6^ with average program sizes of under 400 beneficiaries, successful programs that care for high-need beneficiaries are often small, and are well-integrated into the local health care system, as they focus on a small subset of the population that is high need and accounts for an outsized share of health care costs, at a panel size conducive to generating the trust that is a key ingredient for successful programs.^18^ The IAH demonstration only required participants to serve a minimum of 200 beneficiaries per year, which enabled many small organizations to participate. High beneficiary minimums like those seen in the SSP, which has a 5000 beneficiary minimum, prevent participation by many locally established small high-needs health care professionals, like HBPC practices, that can and have succeeded under high needs–specific models.

#### Value-Based Payment

Traditional FFS payments cannot support the high touch, interdisciplinary team-based care that is provided under IAH and other high-needs models. Many aspects of team-delivered care management and coordination are not eligible for payment under Medicare FFS, and in aggregate those payments are not enough to support the interprofessional team and the longer, more intensive visits that the high-needs population requires. Value-based payment methods, like the IAH shared savings payment incentive, allow participants to benefit when they help patients avoid unnecessary care like preventable hospitalizations and emergency department visits. These shared savings provide greater reimbursement than FFS revenue for health care professionals who deliver high quality care and offer greater flexibility to provide non–face-to-face care management and coordination.

#### Full Panel Coverage for Practices Providing Care for Patients With High Needs

Currently only 50% to 60% of a typical HBPC practice meets the IAHQ or the ACO REACH High Need criteria.^6^ This means that the remaining 40% to 50% of the practice’s patients are not part of an accountable care relationship. In order to preserve a program option specific to patients with high needs while achieving the CMS goal of all beneficiaries in an accountable care relationship by 2030,^19^ CMS could structure programs with multiple care plans (commonly referred to as “tracks”) for beneficiaries at different risk levels, or allow clinicians’ frail patients who do not rise to the high-need threshold to participate in another value-based model that covers those patients who do not qualify.

### Limitations

There are limitations to these analyses. Due to JFI licensing and data restrictions, our MA analysis used an alternative claims frailty index, which we set to a JFI-equivalent threshold, although there might be some residual instrument-level measurement error. Coding intensity in MA may bias the CFI upwards, although the impact of upcoding bias would be to overstate the IAH-qualified share of MA beneficiaries, narrowing the difference with TM. Earlier MA encounter data had problems with completeness, although those have substantially improved since 2018. Even with improved completeness, incomplete MA data in some contracts may undercount IAH-qualifying utilization, such as skilled nursing facilities or Home Health. We had only a cross-sectional sample (2021) for IAH-qualified MA prevalence, while multiple years would better define the trend in high-needs MA enrollment. A few MA plans have invested in home-based primary care models through programs such as Landmark, supporting robust HBPC programs in MA for some localities and some patients. We did not assemble an IAH-qualified incidence cohort for MA, so that lower IAH-qualified beneficiary prevalence might have been partly due to reduced hospital and postacute care qualifying utilization under MA. We only had a cross-sectional comparison of TM to MA, while we would need a longitudinal analysis to directly measure disproportionate non-IAH–qualified beneficiary enrollment in MA.

## Conclusions

Despite these limitations, the lower prevalence of IAH-qualified beneficiaries in MA and the growth of the number of IAH-qualified beneficiaries in TM implies TM must have value-based payment models that sustainably address the needs of high-need beneficiaries and the health care professionals who take care of them. While CMS is doing further testing of many of the features required by high-needs beneficiaries and health care professionals in the High Needs ACO REACH track, built on the success of the IAH model, that demonstration expires in 2026.^20^ The growing share of high-need beneficiaries in TM indicates the need for a sustainable, long-term high-needs program within TM. A permanent high-needs program, whether standalone or as an option in the Medicare Shared Savings Program, would ensure that the growing population of frail, high-need beneficiaries in TM can continue to receive personalized, high-quality care.

## Notes

Supplement 1. **eTable 1.** IAH Demonstration Cost Outcomes Y1-8 **eTable 2.** IAH Qualified Prevalence in TM and MA by State **eTable 3.** IAH Qualified TM Cost and Share of Cost by State **eAppendix 1.** Alignment of CMMI High Needs Criteria With IAH Qualification Using Either JFI 6+ or CFI = .20 **eAppendix 2.** MA Benchmark Implications of TM Risk Adjustment Using Prospective HCC Model

Supplement 2.
Data Sharing Statement
