# Supplementary material for: Prevalence of Independence at Home–Qualifying Beneficiaries in Traditional Medicare, 2014-2021
Source: JAMA Netw Open. 2024 Jul 11;7(7):e2421102. doi: 10.1001/jamanetworkopen.2024.21102 (PMC11240187; doi:10.1001/jamanetworkopen.2024.21102)
Supplement: Supplement 1. — eTable 1. IAH Demonstration Cost Outcomes Y1-8 eTable 2. IAH Qualified Prevalence in TM and MA by State eTable 3. IAH Qualified TM Cost and Share of Cost by State eAppendix 1. Alignment of CMMI High Needs Criteria With IAH Qualification Using Either JFI 6+ or CFI = .20 eAppendix 2. MA Benchmark Implications of TM Risk Adjustment Using Prospective HCC Model [file jamanetwopen-e2421102-s001.pdf]

Supplemental Online Content

Lally T, Johnson E, Deligiannidis KE, et al. Prevalence of independence at home-qualifying beneficiaries in traditional Medicare, 2014-21. *JAMA Netw Open.* 2024;7(7):e2421102. doi:10.1001/jamanetworkopen.2024.21102

eTable 1. IAH Demonstration Cost Outcomes Y1-8

eTable 2. IAH Qualified Prevalence in TM and MA by State

eTable 3. IAH Qualified TM Cost and Share of Cost by State

eAppendix 1. Alignment of CMMI High Needs Criteria With IAH Qualification Using Either JFI 6+ or CFI = .20

eAppendix 2. MA Benchmark Implications of TM Risk Adjustment Using Prospective HCC Model

This supplemental material has been provided by the authors to give readers additional information about their work.

© 2024 Lally T et al. *JAMA Network Open*.

Table E1 IAH Demonstration Savings Results: Announced Savings, Evaluation Cohort Savings, and the Change in Savings from the Payment Incentive

	Y1 N=17	Y2 N=15	Y3 N=15	Y4 N=14	Y5 N=14	Y6 N=11	Y 7 N=10	Y8 N=7	Total Y1-8
Beneficiaries	8245	10,484	11,382	11,762	12,244	6,354	6,436	3,901	**72,603**
PBPM savings (Actuarial)	$151	$112	$183	$368	$350	$534	$697	$899	$360
**Announced Savings***	**$11,988,796**	**$7,821,374**	**$16,367,180**	**$33,781,498**	**$32,514,007**	**$32,352,432**	**$42,632,064**	**$32,074,022**	**$209,533,168**
PBPY savings	$1,454	$746	$1,438	$2,872	$2,656	$5,092	$6,624	$8,222	$2,886
Eval PBPM savings (IAH-comparison beneficiaries)	$221	$133	$280*	$384*	$432**	$412**	$860**	$703**	$395
Eval Savings (IAH-comparison beneficiaries)	$17,546,516	$9,281,937	$24,980,200	$35,256,576	$40,155,696	$24,941,244	$52,570,080	$25,069,683	$229,801,932
Eval PBPM Change in Savings	$119	$31	$178	$282	$330*	$31	$459*	$320	$201
Eval Change in Savings	$9,448,124	$2,162,808	$12,854,270	$25,442,886	$31,350,990	$3,190,507	$22,648,708	$9,921,292	$117,019,585
**CMS Net Eval Savings**	**$5,878,493**	**$3,959,594**	**$17,250,417**	**$27,161,566**	**$33,299,873**	**$13,891,161**	**$34,079,246**	**$12,599,594**	**$148,119,944**
CMS Announced Savings									
MPR Evaluation: Difference in Differences or Change in Savings									
MPR Evaluation Cohort : Difference from Comparison Beneficiaries									
p<0.05	*								
p<0.01	**								

Table above combines annual CMS savings announcements (Actuarial savings), with Evaluation reports Y4-8 (Evaluation Change in Savings), and with Evaluation cohort results taken from Appendices in the Evaluation reports (Evaluation Savings), detailed in

Deligiannidis KE, Boling P, Taler G, Leff B, Kinosian B. Independence at Home: After 10 years of evidence, it's time for a permanent Medicare program. *J Am Geriatr Soc*. 2023; 71(9): 3005-3009. doi:10.1111/jgs.18386

The difference between the Evaluation cohort results and the Evaluation reports is that the Evaluation used a difference in differences approach, so could only address the question of the impact of the IAH payment incentive on ***changing*** the difference between IAH qualified HBPC patients and their controls, as over 70% of participants were receiving HBPC before entry into the

© 2024 Lally T et al. *JAMA Network Open*.

demonstration. The Evaluation cohort results compares the demonstration beneficiaries with their matched control cohort, without removing the prior effect of HBPC (the central part of the IAH care model). The CMS net savings are the Evaluation cohort savings, less the shared savings paid to the practices.

© 2024 Lally T et al. *JAMA Network Open*.

Table E-2 2021 TM and MA Population, % IAH, and MA share of Medicare

	TM Population	TM IAH Population	% IAH in TM	MA Population	MA IAH Population	% IAH in MA	MA % in Medicare
United States	30,035,511	3,215,057	10.7%	26,909,116	2,150,038	8.0%	47.3%
Alabama	458,201	55,892	12.2%	541,491	49,438	9.1%	54.2%
Alaska	90,953	4,176	4.6%	2,286	115	5.0%	2.5%
Arizona	386,175	40,554	10.5%	632,647	42,324	6.7%	62.1%
Arkansas	651,410	53,101	8.2%	222,553	19,740	8.9%	25.5%
California	2,739,406	286,127	10.4%	3,062,462	177,317	5.8%	52.8%
Colorado	428,484	34,885	8.1%	442,796	30,154	6.8%	50.8%
Connecticut	299,427	43,100	14.4%	339,040	30,276	8.9%	53.1%
Delaware	54,358	5,632	10.4%	51,680	4,310	8.3%	48.7%
District of Columbia	153,664	16,427	10.7%	23,831	2,438	10.2%	13.4%
Florida	2,066,076	269,220	13.0%	2,443,948	197,227	8.1%	54.2%
Georgia	812,976	82,650	10.2%	854,964	65,576	7.7%	51.3%
Hawaii	110,002	6,182	5.6%	142,813	7,183	5.0%	56.5%
Idaho	189,749	14,207	7.5%	145,883	9,234	6.3%	43.5%
Illinois	1,302,773	150,906	11.6%	780,299	66,169	8.5%	37.5%
Indiana	685,441	73,318	10.7%	525,975	46,444	8.8%	43.4%
Iowa	421,268	36,379	8.6%	181,303	12,582	6.9%	30.1%
Kansas	370,238	38,792	10.5%	140,904	12,681	9.0%	27.6%
Kentucky	472,968	52,150	11.0%	415,236	36,084	8.7%	46.8%
Louisiana	417,717	52,464	12.6%	420,056	36,167	8.6%	50.1%
Maine	163,850	15,356	9.4%	168,810	9,943	5.9%	50.7%
Maryland	749,962	80,998	10.8%	176,141	12,541	7.1%	19.0%
Massachusetts	833,862	112,735	13.5%	401,151	41,599	10.4%	32.5%
Michigan	898,485	101,693	11.3%	1,105,949	96,107	8.7%	55.2%

© 2024 Lally T et al. *JAMA Network Open.*

	TM Population	TM IAH Population	% IAH in TM	MA Population	MA IAH Population	% IAH in MA	MA % in Medicare
Minnesota	430,337	39,995	9.3%	558,648	34,916	6.3%	56.5%
Mississippi	398,982	49,995	12.5%	183,116	16,261	8.9%	31.5%
Missouri	617,065	67,261	10.9%	556,589	49,759	8.9%	47.4%
Montana	167,765	9,772	5.8%	56,034	3,166	5.7%	25.0%
Nebraska	242,907	22,617	9.3%	88,178	7,425	8.4%	26.6%
Nevada	259,021	26,567	10.3%	244,949	17,930	7.3%	48.6%
New Hampshire	201,367	20,833	10.3%	81,726	6,440	7.9%	28.9%
New Jersey	888,874	103,591	11.7%	581,052	45,787	7.9%	39.5%
New Mexico	214,919	15,437	7.2%	186,915	12,486	6.7%	46.5%
New York	1,656,427	191,612	11.6%	1,709,370	121,707	7.1%	50.8%
North Carolina	1,025,431	103,140	10.1%	929,015	77,015	8.3%	47.5%
North Dakota	94,090	7,229	7.7%	31,212	680	2.2%	24.9%
Ohio	1,058,685	120,236	11.4%	1,180,527	119,587	10.1%	52.7%
Oklahoma	465,581	56,509	12.1%	235,276	21,645	9.2%	33.6%
Oregon	387,290	25,602	6.6%	442,799	25,062	5.7%	53.3%
Pennsylvania	1,256,502	147,209	11.7%	1,311,548	135,876	10.4%	51.1%
Rhode Island	87,965	11,221	12.8%	116,163	10,559	9.1%	56.9%
South Carolina	641,376	64,524	10.1%	421,932	39,408	9.3%	39.7%
South Dakota	121,151	8,973	7.4%	48,092	1,303	2.7%	28.4%
Tennessee	668,937	70,723	10.6%	639,395	59,719	9.3%	48.9%
Texas	1,971,350	224,047	11.4%	2,038,933	174,737	8.6%	50.8%
Utah	200,873	18,209	9.1%	185,187	12,352	6.7%	48.0%
Vermont	111,364	9,975	10.3%	30,694	1,832	6.0%	21.6%
Virginia	958,595	98,434	9.0%	462,792	40,911	8.8%	32.6%
Washington	736,141	51,994	7.1%	566,729	29,300	5.2%	43.5%
West Virginia	227,485	24,238	8.7%	190,092	16,443	8.7%	45.5%
Wisconsin	546,093	47,243	10.7%	603,048	41,610	6.9%	52.5%
Wyoming	99,694	6,819	6.8%	6,888	450	6.5%	6.5%

© 2024 Lally T et al. *JAMA Network Open*.

e-Table 3

TM IAH qualified Beneficiary Cost and Share of TM Spending 2021

State	TM Population N	Medicare Payments	IAHQ Beneficiaries N	% TM	% of TM Payments	Total IAH TM0 Payments	IAH PBPY
United States	30,402,759	$349,634,404,950	3,266,773	10.7%	44.4%	$155,304,766,868	$47,541
AL	458,201	$4,900,507,601	55,892	12.2%	44.4%	$2,175,209,128	$38,918
AR	386,175	$3,925,575,134	40,554	10.5%	42.4%	$1,664,264,654	$41,038
AZ	651,410	$6,766,442,465	53,101	8.2%	35.9%	$2,426,934,334	$45,704
CA	2,739,406	$36,848,234,157	286,127	10.4%	47.3%	$17,436,031,244	$60,938
CO	428,484	$4,320,633,501	34,885	8.1%	35.9%	$1,551,527,972	$44,476
CT	299,427	$4,034,235,274	43,100	14.4%	52.2%	$2,106,008,770	$48,863
DC	54,358	$671,936,092	5,632	10.4%	46.9%	$315,015,637	$55,933
DE	153,664	$1,804,512,498	16,427	10.7%	44.2%	$797,070,911	$48,522
FL	2,066,076	$25,441,662,788	269,220	13.0%	46.4%	$11,807,679,824	$43,859
GA	812,976	$8,861,872,667	82,650	10.2%	41.4%	$3,668,421,829	$44,385
HI	110,002	$950,503,332	6,182	5.6%	33.2%	$315,489,413	$51,034
IA	421,268	$4,218,308,558	36,379	8.6%	35.3%	$1,490,616,936	$40,975
ID	189,749	$1,756,588,179	14,207	7.5%	33.4%	$585,902,699	$41,240
IL	1,302,773	$15,345,857,812	150,906	11.6%	46.4%	$7,127,105,582	$47,229
IN	685,441	$7,657,393,724	73,318	10.7%	43.7%	$3,349,702,626	$45,687
KS	370,238	$4,072,014,348	38,792	10.5%	41.9%	$1,706,466,482	$43,990
KY	472,968	$4,861,168,545	52,150	11.0%	44.3%	$2,155,476,919	$41,332
LA	417,717	$4,881,429,420	52,464	12.6%	48.3%	$2,355,891,789	$44,905
MA	833,862	$10,575,430,957	112,735	13.5%	51.4%	$5,436,954,298	$48,228
MD	749,962	$9,917,868,208	80,998	10.8%	46.3%	$4,595,356,061	$56,734
ME	163,850	$1,571,075,333	15,356	9.4%	40.3%	$633,298,745	$41,241
MI	898,485	$9,998,335,280	101,693	11.3%	44.8%	$4,481,261,609	$44,067
MN	430,337	$4,794,665,807	39,995	9.3%	38.4%	$1,840,895,056	$46,028

© 2024 Lally T et al. *JAMA Network Open*.

MO	617,065	$6,716,102,668	67,261	10.9%	43.1%	$2,896,915,175	$43,070
MS	398,982	$4,538,669,116	49,995	12.5%	47.1%	$2,137,695,653	$42,758
MT	167,765	$1,517,343,693	9,772	5.8%	28.2%	$427,901,756	$43,789
NC	1,025,431	$10,363,914,346	103,140	10.1%	40.6%	$4,209,492,136	$40,813
ND	94,090	$955,110,102	7,229	7.7%	33.5%	$319,545,014	$44,203
NE	242,907	$2,667,236,282	22,617	9.3%	38.6%	$1,029,276,309	$45,509
NH	201,367	$2,058,232,445	20,833	10.3%	44.5%	$916,227,575	$43,980
NJ	888,874	$11,997,983,819	103,591	11.7%	47.5%	$5,703,838,689	$55,061
NM	214,919	$1,922,169,690	15,437	7.2%	35.9%	$689,732,219	$44,680
NV	259,021	$3,082,672,505	26,567	10.3%	47.9%	$1,477,550,296	$55,616
NY	1,656,427	$23,132,860,427	191,612	11.6%	48.2%	$11,155,289,738	$58,218
OH	1,058,685	$11,404,525,603	120,236	11.4%	46.2%	$5,268,776,034	$43,820
OK	465,581	$5,443,090,469	56,509	12.1%	46.4%	$2,527,940,466	$44,735
OR	387,290	$3,528,081,809	25,602	6.6%	32.7%	$1,153,404,330	$45,051
PA	1,256,502	$13,878,197,929	147,209	11.7%	46.5%	$6,450,066,152	$43,816
RI	87,965	$946,116,883	11,221	12.8%	49.5%	$467,875,163	$41,696
SC	641,376	$6,587,079,999	64,524	10.1%	41.2%	$2,714,907,156	$42,076
SD	121,151	$1,245,031,179	8,973	7.4%	32.9%	$409,685,402	$45,658
TN	668,937	$6,782,273,142	70,723	10.6%	42.5%	$2,883,873,821	$40,777
TX	1,971,350	$24,545,953,292	224,047	11.4%	46.6%	$11,445,371,097	$51,085
UT	200,873	$2,071,930,881	18,209	9.1%	37.6%	$778,401,734	$42,748
VA	958,595	$9,592,398,541	98,434	10.3%	42.4%	$4,070,237,762	$41,350
VT	111,364	$847,852,779	9,975	9.0%	42.4%	$359,832,930	$36,073
WA	736,141	$6,941,335,340	51,994	7.1%	33.9%	$2,354,680,582	$45,288
WI	546,093	$5,386,782,078	47,243	8.7%	38.1%	$2,054,813,333	$43,495
WV	227,485	$2,273,460,305	24,238	10.7%	45.4%	$1,032,515,928	$42,599
WY	99,694	$1,031,747,952	6,819	6.8%	33.6%	$346,337,901	$50,790

© 2024 Lally T et al. *JAMA Network Open*.

E-Methods A: Alignment of JEN Frailty Index and Kim Claims Frailty Index to identify IAH qualified Medicare Beneficiaries in TM and MA.

We use two different frailty indices, at two different time points: the JEN frailty index in 2014[1], and the JEN frailty index (JFI) and the Kim Claims Frailty (CFI) index in 2021. The use of the CFI for the Medicare Advantage cohort was necessitated due to the proprietary JFI license limitations, precluding Dr. Yao's access.

To calibrate the instrument thresholds, we used the 2021 CMMI High Needs ACO REACH definition of a high needs population, which we had previously shown was equivalent to an IAH qualified population in 2021, using a JFI threshold of 6+ in 100% Traditional Medicare claims data[2].

For computation ease, we used the 5% flag in the Medicare claims for further calibration analyses.

We replicated the 2021 HN-IAH qualified comparison in the 5% file. We then identified the CFI threshold which would identify the same share and characteristics of the TM population as IAH Qualified. That threshold was a CFI of .20, with similar differences from HN ACO REACH to the IAH qualified identified by a JFI 6+. (E-Table A-1)

Medicare 5% Sample*	HNPQ	IAHQ 6+	IAHQ CFI .20
Number of Patients	221,742	164,482	160,251
% of Total Patients	14.05%	10.42%	10.15%
Dual Eligible (January)	32%	26%	27%
HCC v24 - Mean Score (End of Year)	3.88	3.54	3.58
CFI - Mean Score (End of Year)	0.26	0.28	0.29
JFI - Mean Score (End of Year)	7.20	7.54	7.53
CCW AD/Dementia	31%	39%	41%
HCC 51/52 Dementia	23%	28%	30%
Death in Year	19%	18%	18%
FFS Follow-up Months (Mean)	10.71	10.86	10.83
Annual Total Medicare Payments (Mean)	$45,178	$47,805	$47,781
Total Medicare Payments (PBPM)	$4,218	$4,403	$4,413
Inpatient Episodes (Rate per 100 Months)	9.65	10.94	10.95
Long Stay NF Utilization in Year	7%	7%	8%

We also compared the subset of patients identified by either JFI 6+ or CFI=.20 who did not overlap with the HN population. Those patients are also nearly identical. (E-Table A-2)

© 2024 Lally T et al. *JAMA Network Open*.

Medicare 5% Sample*	IAHQ Only (Not HNPQ)	IAHQ Only (Not HNPQ)
	IAHQ 6+	IAHQ CFI .20
% of Total Patients	3.68%	3.49%
Dual Eligible (January)	17%	18%
HCC v24 - Mean Score (End of Year)	1.70	1.73
CFI - Mean Score (End of Year)	0.23	0.24
JFI - Mean Score (End of Year)	6.33	6.25
CCW AD/Dementia	30%	33%
HCC 51/52 Dementia	20%	22%
Death in Year	7%	8%
FFS Follow-up Months (Mean)	11.49	11.46
Annual Total Medicare Payments (Mean)	$21,066	$20,760
Total Medicare Payments (PBPM)	$1,833	$1,811
Inpatient Episodes (Rate per 100 Months)	4.39	4.29
Long Stay NF Utilization in Year	3%	4%
% of Total IAHQ	35%	34%

We then compared the CFI .20 population in MA with the TM JFI 6+ IAH qualified population and the general TM population. (E-Table A-3)

VARIABLE_DESCRIPTION	TM Population	TM IAHQ (JFI 6+)	MA CFI=.20
Male	45%	41%	38.4%
Female	55%	59%	61.6%
Age - Mean	72.54	78.04	75.80
Age less than 75	59%	36%	42.8%
Age 75-84	28%	34%	35.6%
Age 85+	12%	30%	21.6%
White	83%	84%	74.2%
NonWhite	17%	16%	25.8%
Original Entitlement=Age/Elderly	79%	76%	
Original Entitlement=Disabled and/or ESRD	21%	24%	
Dual Medicare/Medicaid	17%	26%	
HCC v24 - Mean (Median, IQR)	1.33 (0.84, 0.47-1.56)	3.54 (2.99, 1.84-4.71)	
CFI - Mean (Median, IQR)	0.17 (0.15, 0.11-0.19)	0.28 (0.27, 0.22-0.33)	29.0%
JFI - Mean (Median, IQR)	4.02 (4, 2-6)	7.54 (8, 6-9)	
Death in Year = Yes	5%	18%	
Total PBPM	$989	$4,403	
CCW AD/Dementia		39%	29.5%

© 2024 Lally T et al. *JAMA Network Open*.

The MA IAH qualified population is more female, younger, more non-White, and with lower prevalence of dementia than the TM IAH qualified population.   Importantly, for concerns about upcoding in MA HCC scoring affecting the CFI calculation, the mean CFI score is equivalent between MA and TM (.29), although the differential share of patients with dementia (39% TM vs 30% MA) suggests a different mix of conditions contributing to the frailty score in the two populations.

1. Orkaby AR, Huan T, Intrator O, et al. Comparison of claims based frailty indices in US veterans 65 and older for prediction of long-term institutionalization and mortal-ity.J Gerontol A Biol Sci Med Sci. 2023;78:glad157. doi:10.1093/gerona/glad157

2. Taler G, Boling P, Deligiannidis KE, Kubisiak J, Lee A, Kinosian B. High needs criteria in High Need Accountable Care Organization Realizing Equity, Access, and Community Health inequitably limits access to equally high-need Medicare beneficiaries. J Am Geriatr Soc. 2023 Oct 29. doi: 10.1111/jgs.18651. Epub ahead of print. PMID: 37898982.

© 2024 Lally T et al. *JAMA Network Open*.

E-Method B: MA benchmark implications of TM risk adjustment using prospective HCC model

MA benchmarks are based on the risk-adjusted Fee-for-Service (FFS) population. To account for differential case mix between FFS (or TM) and MA, the costs for the FFS population are risk-adjusted. If high needs patients are over-represented in TM, the risk adjustment model must adequately account for their extra risk, or the benchmark will be over-stated (due to the inadequately accounted for costs of the high needs—in this case, IAHQ—population). Current prospective risk adjustment models over-estimate cost for low-risk patients, and under-estimate cost for high risk (i.e., IAHQ) patients[1]. If the IAHQ share in TM is larger than in MA, costs will be higher (after the inadequate risk adjustment), so will inflate the MA benchmark. If IAHQ have the same impacts in MA, then of the $348B paid to MA in 2021, the benchmark would be based on 11% rather than 8% IAHQ. That 3% case mix mismatch would represent 12.1% of MA spending (44%/10.7% in TM x 3%), or $42B. The unadjusted prospective HCC model underestimates the cost for IAHQ beneficiaries by 15%, which would be $6B ($42B x .15), to compare with the $17B estimated by MedPAC for all MA overpayments excluding case mix[2]. This would suggest that the MedPAC estimate of MA overpayment is 27% lower than the actual overpayment.

1. Actuarial Research Corporation "Independence at Home Demonstration Revised Actuarial Shared Savings Methodology" Specifications, May 25, 2021 https://www.cms.gov/priorities/innovation/media/document/iah-rev-act-meth-specs
2. MedPAC "Medicare Program Status Report" March 15, 2023 https://www.medpac.gov/wp-content/uploads/2023/03/Ch11_Mar23_MedPAC_Report_To_Congress_SEC.pdf

© 2024 Lally T et al. *JAMA Network Open*.
