# Supplementary material for: Prevalence of Independence at Home–Qualifying Beneficiaries in Traditional Medicare, 2014-2021
Source: JAMA Netw Open. 2024 Jul 11;7(7):e2421102. doi: 10.1001/jamanetworkopen.2024.21102 (PMC11240187; doi:10.1001/jamanetworkopen.2024.21102)
Supplement: Supplement 2. — Data Sharing Statement [file jamanetwopen-e2421102-s002.pdf]

Data Sharing Statement

Data
Data available: No

Additional Information
Explanation for why data not available: Data in VDRC, can only export tabular results. Full tabular results will be available in the online appendix.
